# Use of rasch methodology to develop a short version of the Health Related Quality of life for Eating Disorders questionnaire: a prospective study

**DOI:** 10.1186/1477-7525-8-29

**Published:** 2010-03-18

**Authors:** Carlota Las Hayas, Jose M Quintana, Jesús A Padierna, Amaia Bilbao, Pedro Muñoz

**Affiliations:** 1 CIBER Epidemiology and Public Health, Research Unit 9th floor, Hospital Galdakao - Usansolo, B° Labeaga s/n, Bizkaia 48960, Spain; 2 Department of Psychiatry, Hospital Galdakao - Usansolo, B° Labeaga s/n, Bizkaia 48960, Spain; 3 Basque Foundation for Health Innovation and Research (BIOEF) - CIBER Epidemiology and Public Health, Plaza Asua n°1, Sondika 48150, Bizkaia, Spain; 4 Department of Psychiatry, Ortuella Mental Health Center, Av Del Minero n°1, Ortuella, Bizkaia 48960, Spain

## Abstract

**Background:**

To confirm the internal structure of the Health Related Quality of Life for Eating Disorders version 2 questionnaire (HeRQoLEDv2) and create and validate a shortened version (HeRQoLED-S).

**Methods:**

324 patients with eating disorders were assessed at baseline and one year later (75.6% of whom responded). We performed a confirmatory factor analysis of the HeRQoLEDv2 using baseline data, and then a Rasch analysis to shorten the questionnaire. Data obtained at year one was used to confirm the structure of the HeRQoLED short form and evaluate its validity and reliability.

**Results:**

Two latent second-order factors -- social maladjustment and mental health and functionality -- fit the data for the HeRQoLEDv2. Rasch analysis was computed separately for the two latent second-order factors and shortened the HeRQoLEDv2 to 20 items. Infit and outfit indices were acceptable, with the confirmatory factor analysis of the HeRQoLED short form giving a root mean square error of approximation of 0.07, a non-normed fit index and a comparative fit index exceeding 0.90. The validity was also supported by the correlation with the convergent measures: the social maladjustment factor correlated 0.82 with the dieting concern factor of the Eating Attitudes Test-26 and the mental health and functionality factor correlated -0.69 with the mental summary component of the Short Form-12. Cronbach alphas exceeded 0.89.

**Conclusions:**

Two main factors, social maladjustment and mental health and functionality, explain the majority of HeRQoLEDv2 scores. The shortened version maintains good psychometric properties, though it must be validated in independent samples.

## Background

Eating disorders (ED) affect millions of people worldwide. Since the earliest publications focusing on quality of life among individuals with an ED [1-8] it has been shown that they have a high degree of impairment in various areas of health-related quality of life (HRQoL). Most of these early studies used generic tools to assess the impact of an ED on physical, mental, and social factors [9]. However, these generic tools did not include specific questions probing how the ED affected these factors which, in most cases, limited the interpretation of the results [10].

The first HRQoL instruments specific to individuals with an ED were published almost simultaneously in 2006 and 2007 [10-14]. We developed one of these, the Health Related Quality of Life for Eating Disorders version 2 (HeRQoLEDv2) questionnaire [13,14], a tool with good validity and reliability. One limitation of this 55-question instrument is that it requires a considerable amount of time to complete. We subsequently decided to develop a shorter version. Some techniques for shrinking the size of questionnaires arise from item response theory (IRT) [15-17], with Rasch analysis being a useful approach. The rationale that makes Rasch models useful as a method to shorten the size of a questionnaire is that they can be employed to assess the unidimensionality of questionnaires, and remove items that disrupt this unidimensionality, identify degrees of trait severity and remove those items that overlap in severity level [18]. In addition, it does not require large samples sizes for adequate parameter estimation [19].

The objectives of the current study were to confirm a hypothesized internal structure of the HeRQoLEDv2, create a shortened version of this questionnaire (HeRQoLED-Short form), and then confirm the structure of the shortened version and examine its validity and reliability. We hypothesized that the first-order factors of the HeRQoLEDv2 could represent two second-order latent traits: "social maladjustment" and "mental health and functionality." We tested this hypothesis in the present study.

## Methods

### Participants

Our detailed selection criteria have been described elsewhere [13,14]. Briefly, the population consisted of ED patients being treated by four collaborating psychiatrists, experts in ED, working in three different mental health services in the province of Bizkaia, Spain. Diagnosis of an ED was performed by psychiatrists attending the patient if the patient met the diagnostic criteria for an ED established by the Diagnostic and Statistical Manual of Mental Disorders-IV [20].

Patients were excluded from the study if they had any serious multiorganic or psychotic disorder that could prevent adequate completion of the materials. To be included in the study, a patient had to participate in the investigation in an informed and voluntary way. The tenets of the Declaration of Helsinki were followed, and the study gained approval from the hospital's ethics committee.

Three questionnaires -- the HeRQoLEDv2, the 12-item Short Form Health Survey (SF-12), and the Spanish version of the Eating Attitudes Test-26 (EAT-26) -- were mailed to each patient's home address soon after recruitment, which we define as time 1 (T1). Those who did not respond in a timely fashion were sent reminders after 15 days and 30 days. The same questionnaires were mailed to patients one year later, which we define as time 2 (T2). As before, those who did not respond in a timely fashion were sent reminders after 15 days and 30 days.

Data from the T1 sample were used to perform confirmatory factor analysis (CFA) of the HeRQoLEDv2 followed by Rasch analysis. The T2 data were used to perform the CFA, validity, and reliability analyses of the shortened version.

### Materials

Sociodemographic data were collected from each participant. In addition, each participant completed three self-administered instruments related to HRQoL and ED:

The HeRQoLEDv2 [13,14] is comprised of 55 items and covering nine domains: symptoms, restrictive behaviors, body image, mental health, emotional role, physical role, personality traits, social relations, and binges. The scores in each domain are converted into a range from 0 to 100, with higher scores indicating a worse perception of HRQoL.

The SF-12 [21,22] is a short generic survey of health status that can be summarized in two subscales: the physical component summary and the mental component summary. Values range from 0 to 100, with higher values indicating better health perception.

The Spanish version of the EAT-26 [23] was used as a measure of general eating disorder pathology. This test is composed of three factors -- dieting concern, bulimia and food preoccupation, and oral control -- and a total score. Its overall values range from 0 to 78, with higher scores indicating greater ED symptomatology.

### Statistical analysis

#### Confirmatory factor analysis of the HeRQoLEDv2

The HeRQoLEDv2 had previously been submitted to an exploratory factor analysis to elucidate the way in which items relate to each other and with the hypothesized factors. Following this validity study [13], we are now able to take a step further and hypothesize an internal structure of the HeRQoLEDv2 items and submit that structure to a confirmatory factor analysis. We excluded binges and symptoms domains from the model because binges domain was an independent domain and the symptoms domain is a list of symptoms rather than a proper measurement scale. A second-order CFA composed of a measurement model and a structural model was performed. We hypothesized a measurement model consisting of seven first-order factors: restrictive behaviors (6 items), body image (8 items), social relations (5 items), mental health (9 items), emotional role (4 items), physical role (4 items), and personality traits (4 items). These seven first order factors could be associated to two second-order latent traits: "social maladjustment" and "mental health and functionality". Based on both the content of the items from the following three first order factors "restrictive behaviours", "body image" and "social relations" and based on the literature, we believed that these three factors shared a common aspect: the impact of having an ED on the socio-cultural life. This impact is manifested in the way of feeding oneself, favouring the increase of restrictive behaviours and of feelings of body dissatisfaction [24]. Also a recent study showed that families of individuals with ED perceived serious difficulties in their interpersonal relationship with the affected one [25].

We also hypothesized that the mental health and functionality of individuals with an ED would affect their scores in the first-order domains of "physical role", "emotional role", "mental health", and "personality traits". The mental health and functionality of ED individuals tend to be represented by a combination of high perfectionist traits, low self-efficacy feelings, stress due to feeling overweight and depressive symptoms [26-28]. All of these traits and feelings are part of the content of the selected first order domains.

We further hypothesized that "social maladjustment" and "mental health and functionality" factors would be correlated given that an individual's mental state is likely to affect his or her social adjustment and vice versa.

Several different fit indices are applicable to these analyses [29,30]. We used the chi-square test divided by degrees of freedom, the results of which had to be less than 2.0 to be acceptable [29]; the root mean square error of approximation, where values of 0.08 or less are acceptable [30]; and the non-normed fit index and comparative fit index, both of which had to be equal to or greater than 0.90 to be satisfactory [29].

Only items that showed factor loadings ≥ 0.40 in the corresponding factor were accepted [29]. The Lagrange multiplier test, which identifies paths or covariances that should possibly be added to the model to improve the fit, was used when the model needed modification.

CFAs were performed with the CALIS procedure of the SAS program (version 8.0) [31].

#### Rasch analysis

The Rasch method was applied to the original version of the HeRQoLED as a means to develop the Health Related Quality of life for Eating Disorders - Short Form (HeRQoLED-S). The Rasch model presumes that a single trait drives item responses [32], so that a person's response to an item that measures a single trait is accounted for by his/her level (amount) on that trait, and not by other factors [33]. The Rasch model assumes that the probability of a given patient responding affirmatively an item is a logistic function of the relative distance between the item location parameter (the difficulty of the item) and the respondent (the ability of the patient), and only a function of that difference [34]. Items along the logit scale are ordered according to its difficulty level; the most difficult ones are at the top and the easiest ones, at the bottom [35]. In our study, items which reflect the highest impact on HRQoL are placed at the top of the continuum and those which reflect the lowest impact are placed at the bottom. We used the polytomous Rasch rating scale model because our response scales are ordinal with six response options. A joint maximum-likelihood estimation process was used to estimate the parameters [36].

Prior to all further analyses, the functioning of rating scale categories was examined for each of the two domains of the HeRQoLED short form. The rating scale categorizations presented to respondents are intended to elicit from those respondents unambiguous, ordinal indications of the locations of those respondents along the latent trait of interest [37]. Therefore the probability of selecting an item response category indicative of better health status should increase as the underlying level of health of the respondent increases [33]. Linacre [37] suggests the following criteria to assess adequate functioning of rating scale categories: (1) More than 10 observations per category (or the findings may be unstable, i.e., non-replicable); (2) A smooth distribution of category frequencies. The frequency distribution is not jagged; (3) Clearly advancing average measures; (4) Average measures near their expected values; (5) Observational fit of the observations with their categories: Outfit mean-squares near 1.0. Values much above 1.0 are much more problematic than values much below 1.0.

Because the condition of unidimensionality is a requirement for using Rasch analysis, we applied the Rasch analysis separately to both social maladjustment and mental health and functionality factors. Unidimensionality was assessed through a principal components analysis (PCA) of the residuals extracted from the Rasch model [18]. A violation of unidimensionality was considered if in addition to the first factor there were other factors with eigenvalues greater than 3 [37]. Apart of the PCA, unidimensionality was assessed through examination of fit statistics. We used two indices of fit, namely the mean square information-weighted statistic (infit) and the outlier-sensitive statistic (outfit). Values between 0.7 and 1.3 for both indices indicate a good fit [38].

We evaluated how well the HeRQoLED - short version differentiates individuals in the measured domains on the basis of the person separation statistic [39] and how well it differentiates items based on the item separation index, which indicates the ability to define a distinct hierarchy of items along the measured variable. A value ≥ 2.0 for this statistic is comparable to a reliability of 0.80 and is acceptable. Correlation of items with the total scale score served to evaluate whether the items correlated in a similar way with the construct being measured [40].

"Item bias" or "differential item functioning" (DIF) occurs when items exhibit different difficulties for different person groups. For a given level of a trait, the probability of endorsing a specified item response should be independent of group membership [32]. For the DIF analysis, we examined whether diagnosis subtype (anorexia nervosa, bulimia nervosa, or eating disorder not otherwise specified) may exert influence on item calibrations in subsamples. DIF analyses were performed independently for the "Social maladjustment scale" and for the "Mental health and functionality scale". Welch *t *gives the DIF significance as a Welch's (Student's) t-statistic. The t-test is a two-sided test for the difference between two means (i.e., the estimates) based on the standard error of the means (i.e., the standard error of the estimates). The null hypothesis is that the two estimates are the same except for measurement error. To establish a noticeable DIF between subsamples, the difference in difficulty of the item between the two groups (DIF contrast) should be at least 0.5 logits. In addition, the Welch *t *should be statistically significant, P < .05 [37].

Residual correlations between items within a scale were examined for local dependency. Correlations > 0.5 between item residuals can indicate that responses to one item may be determined by those to another [41].

Rasch analyses were repeated until we obtained a version that met the criteria, which was named the Health Related Quality of Life for Eating Disorders-Short Form (HeRQoLED-S). Item content was examined for the misfitting items before removal from the scale. Two of the authors of the present study (JAP and CLH) are experts in the field of eating disorders. They jointly decided whether to retain or delete an item based on the clinical importance of the content. Winsteps version 3.37 was used for the Rasch analysis [42].

#### Confirmatory factor analysis of the HeRQoLED-S

A CFA was applied to the shortened version. The hypothesized structural and measurement models were the same as those of the long version. The only difference was that fewer items were assigned to each first-order factor. The same fit indices were also used to assess the goodness of fit.

#### Validity and reliability of the HeRQoLED-S

Based on content similarity between subscales of different questionnaires, we hypothesised the following correlations for the analysis of concurrent validity: The social maladjustment factor would correlate positively and moderately, by means of the Pearson correlation coefficient, with the dieting concern factor of the EAT-26. The mental health and functionality factor, in turn, was hypothesized to correlate negatively and moderately with the mental component summary of the SF-12. The Cronbach alpha index of reliability was calculated for each factor; values above 0.70 were acceptable [43].

## Results

### Participants

A total of 394 ED patients were approached for the study. Of them, 324 ED patients completed the first set of questionnaires (T1). All patients were receiving treatment for their ED at T1 but they differed in ED subtype, severity and time in treatment. We did not filter patients in these regards; therefore we expect that these patients represent the entire spectrum of ED severity. All were asked to complete the same tests again after one year. Of these, 245 patients (75.6%) responded. Most participants were women (96.3% at T1 and 95.1% at T2), with a mean age of 27 years, SD (8.76) at T1. From the baseline sample, 21% patients had been diagnosed with anorexia nervosa, 15% with bulimia nervosa, and 64% with eating disorders not otherwise specified.

#### Confirmatory Factor Analysis of the HeRQoLEDv2

For the CFA, only data from the 262 participants who completed the HeRQoLEDv2 at T1 with no answers missing were used. The hypothesized model described in the Introduction provided satisfactory fit indices after few adjustments. Following the Lagrange multiplier test, two pairs of errors, one belonging to the body image domain and the other to the social relations domain, were allowed to covary. Additionally, the Lagrange multiplier test suggested setting a new causal relationship between the personality traits item "Have you had lack of confidence in your own capabilities?" and the mental health domain item "Have you felt yourself worthless?". This new relation is meaningful given that lack of confidence in one's capabilities may lead an individual with an ED to feelings of worthlessness when facing problems. After these adjustments, the goodness of fit indices for the model were satisfactory (χ^2 ^(*df *= 729) = 1464.67, *P *< .0001; χ^2^/*df *= 2.01; RMSEA = 0.06; NNFI = 0.90 and CFI = 0.90).

**Figure 1:**
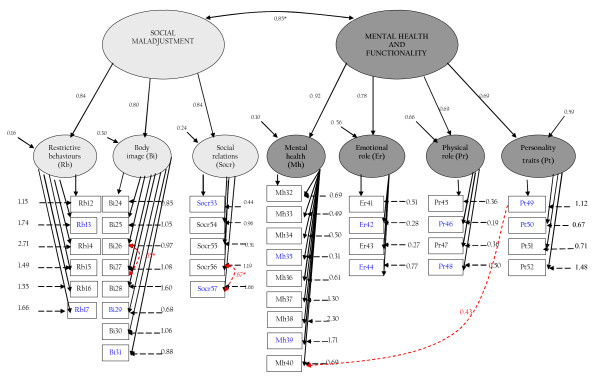
**Path diagram of the resulting structure of the HeRQoLEDv2**. In order to keep the path diagram from becoming overly complex, the lowest and highest factor loadings for each domain are described here: Restrictive behaviors = .49 - .71, Body Image = .70 - .87, Social relations = .57 - .89, Mental Health = .54 - .85, Emotional role = .81 - .94, Physical role = .84 - .95 and Personality Traits = .64 - .84. * Indicates covariances among exogenous variables.

Figure 1 shows the path diagram of the model with the estimated parameter values included.

#### Rasch analysis to obtain the shortened version

Data from all 324 ED patients who responded at T1 were used for the Rasch Rating Scale analysis.

Originally, the social maladjustment domain was composed of 19 items. Nine of them were removed because they showed inadequate fit indices (infit or outfit) or because they overlapped the same level of difficulty as other items. Experts in ED evaluated the importance of the item content before removing the item. The shortened social maladjustment domain consisted of 10 items separated by 0.10 or more logit values. Table 1 shows the characteristics of the measurement level, standard error, infit, outfit, and item total correlations. The level of difficulty is represented by the trait level (*δ*), where high values indicate greater difficulty with social adjustment.

**Table 1:** Rasch model: Item measure, SE, fit statistics and item-total correlations of the social maladjustment domain

		Social maladjustment				
**Item^a^**	**Content**	**δ**	**SE**	**Infit**	**Outfit**	***r*_t_**

(1) RB12	Do you fast for a day, although you feel hungry?	1.54	0.07	1.17	0.92	0.58

(2) RB13	Do you skip some meals, although you feel hungry?	0.56	0.05	1.33	1.12	0.69

(3) RB15	Do you avoid eating with others?	0.48	0.05	1.12	1.19	0.61

(10) SOCR56	Do you think that your eating habits negatively affect your personal relationship or the possibility of finding one?	0.23	0.05	1.21	1.16	0.63

(9) SOCR54	To what extent do your concerns about eating negatively affect your family relationship (talking less, discussing more, diminished confidence?)	0.12	0.05	1.03	1.17	0.62

(8) BI28	Do you avoid situations in which others can see your body, for example, in the gym, the pool, or on the beach?	-0.01	0.05	1.26	1.23	0.70

(4) BI24	In general, do you feel fat, despite the fact that other people (family, friends, doctors, etc.) tell you otherwise?	-0.40	0.05	0.83	0.80	0.81

(5) BI25	Do you think that some parts of your body, for example, hips, waist or thighs, are too big or wide compared with the rest of your body?	-0.52	0.05	0.95	0.89	0.78

(6) BI26	Do you worry about your weight?	-0.92	0.05	0.82	0.80	0.79

(7) BI27	Do you worry about possibly gaining weight?	-1.09	0.06	0.77	0.84	0.78

Every question has a response scale of 6 ordinal options, being 0 = Never and 5 = Always.

δ = Level of severity of the social maladjustment factor. Higher values indicate higher severity; SE = standard error;*r*_t _= correlation between item and total measured social maladjustment level based on the Rasch calibrated item scores and total scores.

^a ^The numbers in parentheses reflect the current item location in the shortened version.

This English translation has not been validated linguistically. We provide an approximate translation of the Spanish items into English.

Four items of the social maladjustment domain did not comply with all the requirements for adequate functioning of rating scale categories. Specifically, fewer than 10 participants had endorsed the response category "Almost always" in the item RB12 "Do you fast for a day although you feel hungry". We combined adjacent categories "almost always" with respondents of "Always" to obtain a robust structure of high frequency categories. This combination reproduced satisfactory results with an outfit index of 1.3. Items RB15 "Do you avoid eating with others?" and BI27 "Do you worry about the possibility of gaining weight?" showed large outfits in one of their response categories. Response category "Always" of item RB15 presented an outfit index of 2.1. After combining respondents of adjacent categories "always" and "almost always", the outfit index reduced to 1.5. For the item BI27 the category response "never" presented an outfit index of 2.6. After combining this response category with the adjacent category of "almost never" the outfit value reduced to 1.4. The fourth problematic item is SOCR54 "Do you think that your eating habits negatively affect your family relationship?" which presented a large outfit (OUTF MNSQ = 1.9) for response category 3 "several times" but not for the remaining of the response categories. Combining adjacent categories was not a good approach since the resulting merged response category would count with an excessive number of respondents. We decided to leave the item as it was.

Unidimensionality was supported since the PCA of the residuals did not give additional factors with eigenvalues exceeding 3.00. Furthermore, the fit indices ranged from 0.77 to 1.30. All the item total correlations were high and homogeneous (see Table 1). Differential item functioning was observed only in one item, BI27 "Do you worry about the possibility of gaining weight?" with a difference slightly higher than 0.5 (DIF contrast = 0.66; p < 0.05) between the anorexia and the bulimia subgroups. For patients with anorexia nervosa, this item was slightly more difficult than for patients with bulimia. Intercorrelations between residuals were all below 0.50 (range -.30 to .47).

**Figure 2:**
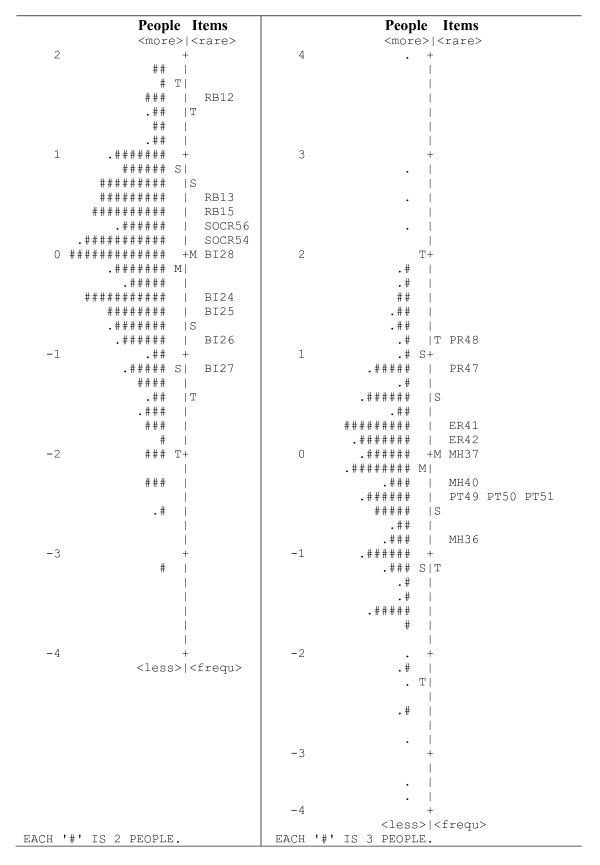
**Person and item map of the social maladjustment and mental health and functionality domains**. Both individuals and items are presented in the same logit scale. Social maladjustment items are presented on the left side, and mental health and functionality items are on the right side. Items are summarized by the acronym of their corresponding first-order factor along with the number they had in the original HeRQoLEDv2. Tables 1 and 2 present a brief description of each question's content. RB = restrictive behaviors; SOCR = social relations; BI = body image; PR = physical role; ER = emotional role; MH = mental health; PT = personality traits.

The final shortened scale of social maladjustment included 10 items. The item locations for the HeRQoLED-S are shown in Figure 2 (left-hand side). The person separation index (2.46) and the item separation index (12.48) exceeded the required value of 2.0, thereby indicating a reliability above 0.80. The total score was transformed to range from 0 to 100 (mean score: 48.8; SD: 23.2).

The mental health and functionality scale originally included 21 items. After performing iterative Rasch analyses and item content analysis, 11 of them were removed because they overlapped or misfit and were not clinically essential. Seven of the 10 remaining items in the scale were separated by 0.10 logit units and 3 of which were separated by 0.04 logit units (Table 2; Figure 2, right). The 3 overlapping items (Figure 2, right-hand side) were retained because they were considered clinically meaningful based on expert opinion and had adequate fit indices.

**Table 2:** Rasch model: Item measure, SE, fit statistics and item-total correlations of the mental health and functionality domain

		Mental health and functionality				
**Item^a^**	**Content**	**δ**	**SE**	**Infit**	**Outfit**	***r*_t_**

(7) PR48	Do you have to stop performing some tasks as a result of your physical problem?	1.16	0.06	1.04	1.05	0.67

(6) PR47	Do you find it difficult to maintain attention as a result of your physical problem?	0.80	0.06	1.11	1.03	0.71

(4) ER41	Do you have to make an extra effort or invest more time than usual as a result of your emotional problems?	0.27	0.06	0.86	0.81	0.76

(5) ER42	Do you accomplish less than you would like to as a result of your emotional problem?	0.17	0.06	0.90	0.85	0.77

(2) MH37	Do you have very sudden mood changes that you find difficult to control?	0.06	0.06	1.17	1.20	0.62

(3) MH40	Do you feel worthless?	-0.35	0.06	0.73	0.73	0.80

(10) PT51	Do you set very high goals and feel dissatisfied if you do not meet them?	-0.39	0.06	1.17	1.14	0.68

(9) PT50	Do you think that you have to do things perfectly or just not to do them at all?	-0.42	0.06	1.24	1.24	0.68

(8) PT49	Do you feel lack of confidence in your own capabilities?	-0.45	0.06	0.87	0.88	0.73

(1) MH36	Do you feel happy?	-0.86	0.06	0.84	0.95	0.64

Every question has a response scale of 6 ordinal options, being 0 = Never and 5 = Always.

δ = Level of severity of the social maladjustment factor. Higher values indicate higher severity; SE = standard error;*r*_t _= correlation between item and total measured social maladjustment level based on the Rasch calibrated item scores and total scores.

^a^The numbers in parentheses reflect the current item location in the shortened version.

This English translation has not been validated linguistically. We provide an approximate translation of the Spanish items into English.

Unidimensionality was supported since the PCA of the residuals did not lead to additional factors with eigenvalues exceeding 3.00. Furthermore, the fit indices ranged from 0.72 to 1.27. The item total correlations were all high and homogeneous, ranging from 0.61 to 0.78.

Only two items of the mental health and functionality domain did not comply with the requirements for adequate functioning of rating scale categories. Specifically, the category response "Always" of item PR48 "Do you have to stop performing some tasks as a result of your physical problem?" presented an outfit index of 2.2. Therefore, we decided to combine this response option with the adjacent category "Almost always". After this combination, the outfit reduced to 1.4. The category response "Never" from the item MH36 "Do you feel happy?" was only reported by 1 participant. Thus, we decided to combine it with the adjacent category response "Almost never" to enlarge the sample. After this combination, the outfit index was -1.58.

Figure 2 (right side) shows the item and person locations along the logit scale. Positive values indicate high levels of mental health disease and dysfunction, whereas negative values indicate low levels of mental health disease and dysfunction.

The person separation index (2.5) and the item separation index (9.7) for this sample also exceeded the required value of 2.00, indicating a reliability of the scale above 0.80. The raw score in this domain was also transformed to range from 0 to 100 (mean = 48; SD = 20.3). Statistically significant DIF contrasts were not observed for any item of the scale.

Intercorrelations between residuals were below 0.50 (range -.29 to .41), except for two items ("Do you have to stop performing some tasks as a result of your physical problem?" and "Do you find it difficult to maintain the attention as a result of your physical problem?") which slightly surpassed this threshold (r = 0.51).

In summary, after applying the Rasch rating scale analysis to the original 40 items (after excluding items from binges and symptoms domains) of the HeRQoLEDv2 we obtained a shortened version of 20 questions divided in 2 factors, 'social maladjustment' and 'mental health and functionality'. This HeRQoLED short version provides separate scores for each factor. Calculating the score in both long and short versions requires summing the response options selected in the factor's items, standardizing the score to range from 0 to 100. In case of missing values we applied the mean imputation method.

#### Confirmatory Factor Analysis of the HeRQoLED-S

Data from the 207 patients who returned questionnaires at T2 without missing answers were used for the CFA of the HeRQoLED-S. The hypothesized model was similar to that of the long version but included only the 20 items accepted after the Rasch analysis. The Lagrange multiplier test was again used. The first pair of errors intercorrelated belonged to two items from the body image domain, and the second to the personality traits domain.

**Figure 3:**
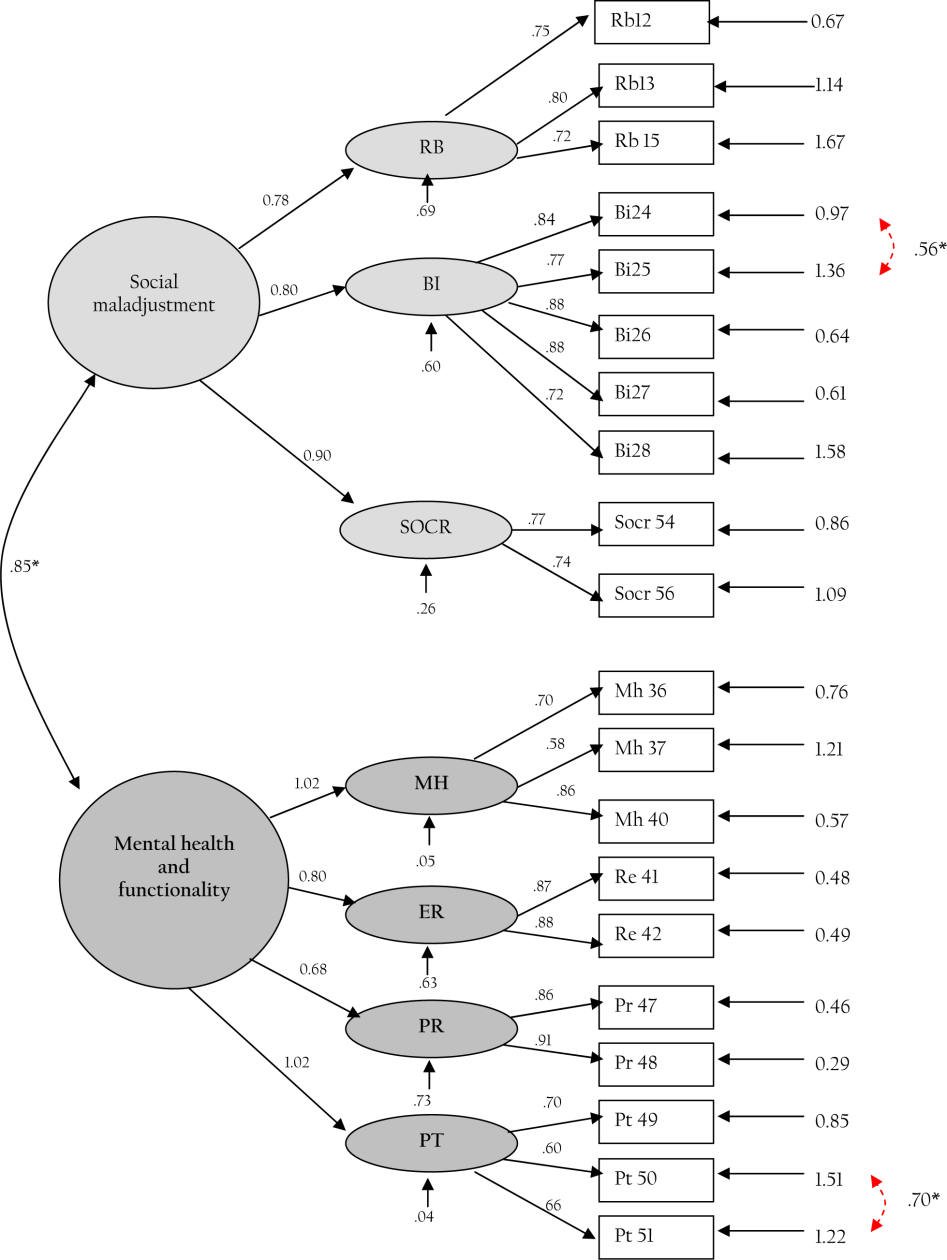
**Confirmed factor structure of the HeRQoLED-S**. RB = restrictive behaviors; BI = body image; SOCR = social relations; MH = mental health; ER = emotional role; PR = physical role; PT = personality traits. * Indicates covariance among exogenous variables.

The factorial structure that resulted after allowing for these covariances between errors proved satisfactory since it resulted in acceptable fit indices (x^2 ^(*df *= 160) = 305.96, P < .0001; x^2^/*df *ratio = 1.9; RMSEA = 0.07; NNFI = 0.93 and CFI = 0.94) and significant factor loadings (Figure 3).

#### Concurrent validity and reliability of the HeRQoLED-S

A data set for the HeRQoLED-S was created using the responses of all 245 patients who completed questionnaires at T2. As hypothesized (Table 3) the social maladjustment factor correlated more strongly with the dieting concern factor of the EAT-26 (r = 0.82, p < 0.001) than with the remaining factors. The mental health and functionality factor of the HeRQoLED-S also correlated higher with the mental summary component of the SF-12 (r = -0.69, p < 0.0001) than with the other factors. The Cronbach alpha was 0.91 for the social maladjustment domain and 0.90 for the mental health and functionality domain.

**Table 3:** Measurement of the concurrent validity and reliability of the HeRQoLED-S

	Social maladjustment	Mental health and functionality
SF-12 PCS	-.27	-.33
SF-12 MCS	-.51	-.69
EAT "Dieting concern" factor	.82	.61
EAT "Bulimia and food preoccupation" factor	.73	.58
EAT "Oral control" Factor	.49	.44

Cronbach alpha	.91	.90

All correlations were assessed using Pearson correlation coefficient. * All correlations were statistically significant at p < .0001; SF-12 PCS = Short-Form-12, Physical Component Summary; SF-12 MCS = Short-Form-12, Mental Component Summary; EAT = Eating Attitudes Test.

## Discussion

This study confirmed the internal structure of a newly developed questionnaire for eating disorders, the 55-question HeRQoLEDv2. We also applied CFA and Rasch analysis to develop a shorter 20-question version, which maintained satisfactory psychometric qualities, and we validated the internal structure of the shortened questionnaire.

Of the three other disease-specific instruments created to date for measuring HRQoL in patients with an ED, only the EDQOL questionnaire [12] has been subjected to a CFA. In that study, the investigators confirmed the structure of a second-order factor, presumed to be the HRQoL construct that explained the relationships between four latent first-order factors. In the current study, CFA of the HeRQoLEDv2 revealed two correlated second-order factors that explained the relationships between seven first-order factors. In theorizing our model, we did not hypothesize an orthogonal structure *a priori *because we assumed that the HRQoL measurement construct included the intercorrelation of physical, mental, and social factors affected by EDs and treatment [44,45].

Validation of the HeRQoLEDv2 using CFA provides the questionnaire with greater construct validity than in the version we previously developed [14]. To perform the CFA, we recruited 262 patients with ED. Although one could argue that this sample size is small considering the length of the questionnaire, it must be noted that it is difficult to recruit patients with ED, so recruiting this amount of participants can be considered as strength of the study more than a limitation. Among the potential statistical drawbacks derived from the sample size are the increase in sampling error, instability, and reduced reliability of factor analysis solutions [46].

A second aim of this study was to use modern analytical techniques to create a shorter version of the HeRQoLEDv2. Various strategies are available for the reduction of questionnaires [15]. We chose to apply the Rasch method, as this technique produces a scale that calibrates items based on their range of difficulty for the target population.

The 20-item HeRQoLED-S that emerged from the Rasch method provided adjustment levels (infit and outfit), unidimensionality, and local independence sufficient to be considered adequate. A slight DIF was observed in only one item. We decided not to remove the item from the questionnaire since it was clinically relevant and presented satisfactory levels of functioning in the other parameters (fit statistics, local dependence, and response scale functioning).

A third aim of the study was to validate the HeRQoLED-S. A CFA applied to the HeRQoLED-S confirmed the goodness of the structure achieved using the Rasch method, as reflected in the obtained fit indices. Other studies have also applied CFA to validate the internal structure of shortened questionnaires [47]. Apart of the hypothesized concurrent correlations between the HeRQoLED-S and specific domains of the EAT-26 and SF-12, the social maladjustment factor of the HeRQoLED-S correlated highly with the second factor of the EAT-26, bulimia and food preoccupation. This latter correlation had not been hypothesized previously. The bulimia and food preoccupation factor contains questions about the control that food exercises over an individual's life and about binges and vomiting. It makes sense that the social maladjustment domain is highly correlated with this factor because individuals who engage in bingeing and vomiting also manifest problems with social adjustment [48,49]. However, our first hypothesis was to correlate the social maladjustment domain with the dieting concern factor because questions pertaining to it inquire about restrictive behaviors and body image, and are more similar in content to those covered by the social maladjustment domain.

We estimated that the shortened form requires approximately 5 to 7 minutes to complete, which is about one-third the time it takes to complete the original HeRQoLEDv2. This is a considerable reduction in time commitment for participants.

One limitation of the HeRQoLED-S is that its items did not cover the entire range of existing difficulties, and gaps in construct difficulty were detected. Although including more items would have helped cover the different levels of construct difficulty, this was not possible because we were working with a predetermined set of items and selected those that provided the best distribution despite the gaps. In addition, although redundant items were identified for mental health and functionality, they were maintained because the scale generally provided good content validity and good fit indices.

Coste et al. [16] have recommended that shortened versions of questionnaires be evaluated psychometrically (particularly with regard to construct validity and reliability) using a new and independent sample. Due to financial limitations and difficulties in recruiting another large sample of patients with an ED, the HeRQoLED-S was validated using the same patient sample as in the follow-up study. We believe this was appropriate given that the T2 sample contained a different number of patients and that the one-year interval since the last contact uses to lead to significant changes in ED symptoms, as some other studies have shown [50,51]. Nevertheless, the same level of validity cannot be obtained from a repeat sample as from a new independent sample. Thus, the shortened HeRQoLED-S must still be validated among different groups of patients with eating disorders.

In conclusion, CFA analysis supports an internal structure of two latent factors of the 55-question HeRQoLEDv2. A short form questionnaire derived from this second order structure, the 20-item HeRQoLED-S, has been developed and validated with modern psychometric techniques that facilitate its use in research and clinical practice. Both versions have demonstrated good reliability and validity. Future applications of HeRQoLEDv2 and HeRQoLED-S using different ED patient samples will yield more evidence about their validity and reliability.

## Competing interests

The authors declare that they have no competing interests.

## Authors' contributions

CLH, JMQ, AP and AB conceived the study. CLH participated in all the phases of the study, design, recruitment of patients, data analysis, interpretation of results, and writing of the manuscript. JMQ supervised the study, participated in the interpretation of results and in the writing of the manuscript. AB designed the study and performed the statistical analysis. AP and PM collaborated actively in patient recruitment, revised the data obtained and reviewed the manuscript writing. All the authors read and approved the final manuscript.
